# Editorial Perspective: The challenge of evaluating ADHD parenting interventions – towards a hybrid approach

**DOI:** 10.1111/jcpp.70069

**Published:** 2025-10-22

**Authors:** Saskia van der Oord, Tycho J. Dekkers, Barbara J. van den Hoofdakker, Manfred Döpfner, Edmund Sonuga‐Barke

**Affiliations:** ^1^ Clinical Psychology Faculty of Psychology and Educational Sciences, KU Leuven Leuven Belgium; ^2^ Accare Child Study Center Groningen The Netherlands; ^3^ Department of Psychiatry University of Groningen, University Medical Center Groningen Groningen The Netherlands; ^4^ Levvel, Academic Center for Child and Adolescent Psychiatry Amsterdam The Netherlands; ^5^ The Research School of Behavioural and Cognitive Neurosciences University of Groningen Groningen The Netherlands; ^6^ Department of Clinical Psychology and Experimental Psychopathology University of Groningen Groningen The Netherlands; ^7^ Department of Child and Adolescent Psychiatry Medical Faculty University and University Hospital of Cologne Cologne Germany; ^8^ Center for Child and Adolescent Cognitive Behaviour Therapy (CEKIP) University Hospital of Cologne Cologne Germany; ^9^ Department of Child & Adolescent Psychiatry Institute of Psychiatry, Psychology & Neuroscience (IOPPN), King's College London London UK

**Keywords:** ADHD

## Abstract

Behavioural parent training (BPT) has been recommended as part of multi‐modal intervention strategies for children with attention‐deficit/hyperactivity disorder (ADHD). The evaluation of its effectiveness, however, is challenging, as meta‐analyses have indicated a discrepancy between effects on most proximal (MPROX) and probably blinded (PBLIND) outcome measures. In this editorial perspective, we provide five hypotheses that may explain this discrepancy. The first three hypotheses assume that the MPROX‐PBLIND discrepancy demonstrates that BPT does not reduce actual ADHD characteristics and that MPROX is picking up a false positive. The final two focus on the limitations of the PBLIND assessments reported in the meta‐analyses and the assumption that they are giving false negatives. We conclude that a hybrid approach, integrating parent ratings and observational measures within a multimethod assessment approach, may provide a path forward. In conclusion, we argue that for parents and clinicians, parent ratings of ADHD characteristics and other parent‐ or child‐rated outcomes, such as mental health, quality of life and general well‐being, are more important than ‘objective’ symptom change, which encourages us to shift the focus from the control of symptoms to the promotion of general functioning and well‐being.

## Are parenting interventions unblindable?

Behavioural parent training (BPT) has been recommended as part of multi‐modal intervention strategies for children with attention‐deficit/hyperactivity disorder (ADHD). BPT for ADHD has a range of intervention targets. First and foremost, it teaches parents behavioural strategies to modify their children's behaviour. At the same time, it aims to strengthen parenting self‐efficacy and mastery, change parental attributions about their child, and improve the parent‐child relationship. Supporting the value of BPT as a treatment for ADHD, RCTs have reported significant effects of small to moderate sizes for both core ADHD characteristics and disruptive behaviours (Doffer et al., 2023; Hornstra et al., 2022).

However, the meaning of these findings is contested. This is because the majority of evidence for such effects comes from ratings by parents who are inevitably ‘unblinded’ (i.e. aware of whether their child received the treatment or the control intervention) and likely to be invested in, and motivated by, a positive outcome of the intervention. This means that reports would be considered by trialists to be subject to a high risk of bias of varying types (e.g. observer biases such as social desirability bias, investment bias, leniency bias and halo effects). Thus, casting doubt on whether reported reductions in children's behavioural difficulties (i.e. the frequency and severity of specific inattentive, impulsive, overactive or oppositional behaviours) reflect ‘real’ changes in behaviours (Seifer, 2005).

The classical solution to these problems of bias is to improve ‘blinding’ – so that the person reporting on changes in the behaviour of a child is unaware of whether that child actually received the intervention or not. There are two ways this can be achieved – blinding by design, which is the gold standard approach, and blinding through measurement. Blinding by design means that recipients of an intervention (in this case, parents and children) are unaware of receiving it. Such an approach allows the use of parent rating scales and is commonly employed in ADHD medication studies and increasingly so in other non‐pharmacological interventions such as neuro‐feedback, through the use of placebo or sham arms, respectively, where treatment and control arms are indistinguishable apart from the presence or absence of the active intervention ingredient. In the case of BPT, this is challenging, as there is generally no trustworthy alternative intervention that does not contain the same ingredients as the active intervention. Attempts have been made to do this, for example with control conditions that focus solely on non‐specific elements of BPT (e.g. a general psychoeducational intervention; Sugaya et al., 2022). However, even then, interpreting such trials is complicated, as parents will almost certainly know the nature and content of the alternative arms, being informed of them for ethical reasons.

## Blinding of measurement – The MPROX vs. PBLIND spectrum

One alternative to blinding by design that has been tried is to *blind by measurement*. This means getting someone else, other than the parent, to assess behaviour change unaware of who received the intervention. The European ADHD Guidelines Group (EAGG) (including at that time ESB, MD and SvdO) addressed this issue head‐on in two systematic reviews of behavioural interventions (among which predominantly parent training interventions) for children with ADHD (Daley et al., 2014; Sonuga‐Barke, Brandeis, Cortese, et al., 2013). The EAGG did this by contrasting two types of trial outcome measures: At one end of the blindedness spectrum are the ‘most proximal’ (MPROX) assessments made by the person closest to the intervention delivery, typically a parent, probably unblinded. At the other end of the spectrum are ‘probably blinded’ (PBLIND) assessments made by someone likely to be unaware of the treatment allocation, with the most blinded being chosen when more than one probably blinded outcome has been measured.

In the original review, the latter was obtained by a single‐session observation in the clinic, at home or at school or by a general rating of the behaviour at school by a teacher who was judged to (probably) be unaware that the child received an intervention, based on the best available evidence (Sonuga‐Barke et al., 2013). While all trials had MPROX assessments, some trials had no assessment that could be considered PBLIND. An additional advantage of this distinction was that it provided the opportunity to compare effects across different types of interventions with different assessments and blinding approaches.

Although the estimates of the effect of behavioural interventions on MPROX and PBLIND assessments of behavioural problems and parenting were quite similar in the EAGG meta‐analyses, they were strikingly different for the assessment of ADHD. Moderate effects (SMD = .36) were found for unblinded MPROX measures, but no effects were found for their PBLIND equivalents (Daley et al., 2014; Sonuga‐Barke et al., 2013). The EAGG concluded that the findings did not support behavioural interventions as frontline treatments for core ADHD characteristics, given the lack of evidence from PBLIND sources and the high risk of bias for the MPROX outcomes. However, the EAGG also highlighted the limitations and ambiguity of the PBLIND measures included in the trials and the possibility that the parent‐rated MPROX measures might be capturing something of clinical significance (Daley et al., 2014; Sonuga‐Barke et al., 2013). Unfortunately, this nuance was lost on many readers who simply came to the conclusion that behavioural treatment, including BPT, doesn't work for ADHD behaviours.

## Why the discrepancy between MPROX and PBLIND assessments of ADHD?

In order to address this over‐simplification regarding the value of BPT for ADHD, we here explore five hypotheses about why there is a discrepancy between MPROX and PBLIND assessments of ADHD.^1^ The first three hypotheses assume that the MPROX–PBLIND discrepancy demonstrates that BPT does not reduce actual ADHD characteristics and that MPROX is picking up a false positive. The final two focus on the limitations of the PBLIND assessments reported in the meta‐analyses and the assumption that they are giving a false negative.

- *Parenting trials produce social desirability effects*: The first hypothesis is that the positive MPROX finding reflects a social desirability bias (i.e. ‘the tendency to give answers that make the respondent good’ towards themselves or others (Paulhus, 1991, p. 17). Parents may want their child to improve (either as impression management or as self‐deception; Paulhus, 1991; Perinelli & Gremigni, 2016) and therefore rate the effects of the intervention more positively. However, it remains a question why this social desirability bias would be specific for MPROX ratings of ADHD behaviour and not for MPROX ratings of parenting and conduct behaviour, especially as socially desirable responding is generally more likely when the behaviour deviates from the social norm (i.e. conduct behaviour) (Perinelli & Gremigni, 2016).
- *BPT changes parents' perceptions*: The second hypothesis is that BPT may change parents' perceptions and/or their tolerance of ADHD behaviours, without actually reducing these behaviours, which would be seen in MPROX but not PBLIND (i.e. leniency bias). This could be due to the emotional investment parents have made in their children and the motivation for therapeutic change, which could positively affect parents' general perception of the child's behaviour after the intervention (i.e. investment bias). Alternatively, BPT may affect parents' tolerance and make parents less critical and hostile, so that changes in perceptual thresholds leading to lower ratings could be an important marker of improved parenting. BPT may also have an effect on the parental (perceived) burden of the child's behaviour or include techniques aimed at increasing parents' individual coping abilities (e.g. parenting stress; Doffer et al., 2023), thereby effectively raising thresholds for what is considered problematic behaviour. It is possible that such changes could be important for the long‐term overall well‐being of the child and an important positive clinical outcome in itself. Of note, also in the diagnostic process of ADHD, clinicians rely primarily on perceptions of behaviour from different informants, with parents as an important source. In that sense, a change in parental perceptions of that behaviour may be meaningful, even if that changed perception is partly due to bias. However, one may also ask whether this should really be considered a bias because a change in parental perceptions of children's problem behaviour may also be an ultimate goal of the intervention.
- *A halo effect of positive change in another outcome*: The third hypothesis is that parents’ ratings of ADHD are affected by a halo bias effect, whereby parents erroneously generalise changes in one aspect of a child's behaviour (e.g. oppositional behaviour) to changes in another aspect of a child's behaviour, when no such changes have occurred (ADHD behaviour). This effect is documented for ratings of ADHD behaviour, although it has been suggested that the effect is more pronounced for teacher ratings than for parent ratings (De Vries, Hartung, & Golden, 2017). Nevertheless, it is conceivable that BPT yields improvements in the child's conduct problems, which casts a halo on parent assessment of ADHD behaviours.
- *Lack of concordance between MPROX and PBLIND measures*: The fourth hypothesis is that BPT reduces ADHD behaviours in the MPROX measurement (most often at home), and parents accurately report this, but that these reductions are not observed using PBLIND assessments. This may be related to the type of rater; a recent meta‐analysis found BPT effects evaluated by teacher ratings were somewhat lower than those using parent ratings but close to zero for observations rated by coders (Doffer et al., 2023). The lack of concordance could also be related to context‐specific effects, as the PBLIND measures for ADHD in the EAGG meta‐analyses were for more than half of the measures drawn from teacher ratings or observations in the classroom, which is a setting/type of assessment for which effects of BPT are perhaps not expected. The obvious antidote to this problem is to use PBLIND assessments in the context in which the BPT is delivered.
- *Lack of reliability/validity of PBLIND observations*: The fifth hypothesis is that even when blinded raters are employed in the home, these assessments *of behaviours* lack *reliability and/or validity*, which could be the reason why blinded outcomes may differ from parent‐rated outcomes. There could be a number of reasons for this. Observational home‐based assessments are carried out over a single relatively short session and are often not obtained during morning and evening routines when most parents report a peak in behavioural difficulties (Koch et al., 2021). Furthermore, behaviour is dynamic, fluctuates throughout the day, can be context‐ and state‐dependent (Rommelse, Bunte, Matthys, et al., 2015) and may not be displayed when children are in a new or arousing situation, such as when an unfamiliar observer is present (e.g. ‘observer reactivity’; Minder, Zuberer, Brandeis, et al., 2018; Rommelse et al., 2015). As such, observational measures may be less valid for capturing the behaviours of interest (i.e. reduced ecological validity). They may lack sensitivity to improvement due to floor effects at baseline and/or fail to capture the dynamic and context‐dependent nature of behaviour. Indeed, reviews show limited validity and problematic psychometric properties of some observational measures of ADHD behaviour, with low temporal stability, often unknown test‐retest reliability and a lack of normative data to assure change can be captured by observational measures of ADHD behaviour (Kelman et al., 2024; Minder et al., 2018). In contrast, for parent ratings of ADHD we mostly do have normative data and test‐retest reliability is usually sufficient. The ecological value added by the parent's framing of their children's behaviour is also likely to be important – parents understand their children's ADHD behaviour better than an observer, which brings positives, as parents may take into account all the experiences they have during a certain period of time to inform their perception of the change of behaviour (e.g. Gomez, Vance, & Stavropoulos, 2018).

## In conclusion

Our analysis illustrates the limitations of both MPROX and PBLIND assessments of BPT‐related changes in children's behaviours. In general terms, these limitations stem from the fact that capturing a child's behaviour, like any human behaviour, is a complex undertaking. It is important to recognise that assessments of behaviour inevitably have both objective and subjective elements – both bring strengths and weaknesses. Parent ratings can be subject to motivational and perceptual biases, while observations may lack reliability and validity. From this perspective, a hybrid approach to assessment, integrating parent ratings and observational measures within a multimethod assessment approach, may provide a path forward. This needs to be informed by a deeper understanding of the meaning and significance of parental ratings and especially a systematic analysis of how these are influenced by biases, motivations and attributions. We also need to improve the observational measures to make them more reliable and ecologically valid. Future research on observational or other ways of assessing behaviour, for example by ecological momentary assessments over multiple days and occasions, novel attention measures (e.g. eye‐tracking glazes) or task‐based measures like actigraphy or continuous performance tests, should focus on exploring their relation to parental perceptions of behaviour and improving their psychometric properties. It is important to place this editorial in a broader context, as many of the issues currently discussed are likely to also apply to assessment of effects of other interventions and to other mental health conditions. For the specific example of BPT for ADHD, many would argue that whether or not BPT ‘really’ improves children's behaviour is neither here nor there and that for parents and clinicians, parent ratings of ADHD characteristics and other parent‐ or child‐rated outcomes, such as mental health, quality of life and general well‐being, are more important. This may also be the case for ADHD treatments more generally, encouraging us to shift the focus from the control of symptoms to the promotion of general functioning and well‐being.

## Ethical considerations

Ethical approval and obtaining participant consent were not applicable for this editorial perspective.

## Data Availability

Data sharing not applicable to this article as no datasets were generated or analysed during the current study.

## Endnote

1 Differences between MPROX and PBLIND measures may be confounded by study characteristics, as effect sizes came from different studies. However, visual inspection of studies including both types of measures suggests similar differences would be found when including only those studies including both MPROX and PBLIND measures.
